# Effects of High‐Frequency rTMS Combined With Physical Exercise on Mood Disorders in Patients With Methamphetamine Use Disorder: A Randomized Clinical Trial

**DOI:** 10.1155/da/6470779

**Published:** 2026-04-07

**Authors:** Kun Wang, Yan Li, Yi Yang, Tingran Zhang, Jiong Luo

**Affiliations:** ^1^ Postdoctoral Station of Shanghai University of Sport, Shanghai, 200438, China; ^2^ College of Liberal Studies (Sports Work Department), Chongqing Industry Polytechnic University, Yubei, Chongqing, 401120, China; ^3^ Research Centre for Exercise Detoxification, College of Physical Education, Southwest University, Chongqing, 400715, China, southwest.edu

**Keywords:** emotional disorder, MA craving, methamphetamine, physical exercise, repetitive transcranial magnetic stimulation

## Abstract

**Background:**

Mood disorders are a severe symptom in patients with methamphetamine (MA) use disorder (MUD) during withdrawal and are closely associated with the risk of relapse. While both standalone repetitive transcranial magnetic stimulation (rTMS) and physical exercise (PE) have shown positive effects on regulating mood disorders, the potential synergistic benefits of their combined use remain unclear. This study aims to investigate the effects of high‐frequency rTMS (10 Hz) combined with PE on mood disorders in patients with MUD and to identify the relevant factors associated with emotional regulation.

**Methods:**

Using a randomized clinical trial design, 54 male patients with MUD were randomly assigned to a PE group, an rTMS combined with PE (rTMS + PE) group, and a control group (CG). All groups received interventions three times per week for a total of 12 weeks (8 weeks of intervention + 4 weeks of follow‐up). The PE group received 10‐min of health education and 35‐min of exercise intervention, the rTMS + PE group received 10‐min of 10 Hz rTMS administered over the left dorsolateral prefrontal cortex (DLPFC) and 35‐min of exercise intervention, and the CG only received 45 min of health education. Measurements for depression, anxiety, MA craving, and blood neurotransmitters were taken from participants at baseline, the 8th week, and the follow‐up period.

**Results:**

(1) Compared with the CG, both intervention groups showed significant reductions in depression, anxiety, and MA craving after the 8‐week intervention, and these improvements were accompanied by marked increases in the blood levels of dopamine (DA), β‐EP, and 5‐HT. Furthermore, these effects persisted for up to 1 month after the intervention concluded. (2) Compared to the PE group, the rTMS + PE group demonstrated significantly lower craving and higher DA levels at the 8th week, with the latter exhibiting superior sustained intervention effects during the follow‐up period (such as negative emotions, craving, DA, and β‐EP). (3) The reduction of negative emotions is not only related to the intervention increasing the release level of neurotransmitters in the blood but also to the decrease in MA craving.

**Conclusion:**

These findings indicate that adding high‐frequency rTMS to moderate‐intensity exercise can produce better therapeutic effects (such as emotion regulation and craving reduction) and increase the sustained impact on the rehabilitation of patients with MUD during the withdrawal period, thus providing a novel strategy for treatment of MUD.

**Trial Registration:** ClinicalTrials.gov identifier: ChiCTR2500105315

## 1. Introduction

Substance use disorder (SUD) refers to the behavioral pattern of an individual’s compulsive seeking and intake of drugs, and it is a process from occasional drug use to compulsive substance use [1]. Emotional regulation disorders are closely related to substance use behavior [2]. On the one hand, SUD can lead to abnormalities in the brain structure and brain function related to emotion regulation in individuals, thereby resulting in emotion regulation deficits in individuals [3, 4]. On the other hand, individuals with higher negative emotional experiences are more inclined to use substances as a coping style to improve mood [5], and this adverse coping style for mood disorders further increases the generation and maintenance of substance use behavior, as well as recurrence after withdrawal [6].

As a typical psychoactive drug of the amphetamine class, methamphetamine (MA) is used at relatively high rates worldwide [7]. Studies have pointed out that long‐term use of MA can lead to mood disorders such as anxiety and depression [8], and these negative emotions are closely related to relapse after withdrawal [9, 10]. In recent years, physical exercise (PE) (especially moderate‐intensity) has been proven to improve the emotion regulation disorder related to withdrawal in patients with MA use disorder (MUD) [11–13], and the improvement of negative emotions is closely related to the reduction of MA craving [14, 15]. Additionally, the impact of PE on withdrawal symptoms (such as mood disorders and cravings) in patients with MUD may be associated with alterations in monoamine neurotransmitter levels in the body [16, 17]. For example, increased levels of neurotransmitters such as dopamine (DA) and serotonin (5‐HT) in the blood may mediate the effects of PE on negative emotions in patients with MUD [15]. However, single‐modality PE interventions often face limitations such as unstable outcomes, difficulty in precisely targeting neural pathways, and short‐lived effects [18, 19], so some researchers have begun incorporating novel methodologies to enable more in‐depth investigations.

Repetitive transcranial magnetic stimulation (rTMS) is a neuromodulation technique characterized by repeated stimulation of specific brain cortical regions, which modulates neuronal electrical activity through magnetic fields generated by coils [20]. Studies have found that non‐invasive brain stimulation techniques, including rTMS, have positive clinical therapeutic effects on patients with SUD [21, 22], especially showing significant therapeutic effects in reducing negative emotions and cravings in patients with MUD [23–25]. For example, after receiving 10 Hz rTMS treatment targeting the left dorsolateral prefrontal cortex (DLPFC), patients with MUD demonstrated significant improvements in emotional attention, decision‐making, executive functions, and craving levels [26–28]. These improvements may be associated with enhanced neuronal activity and increased cortical excitability induced by high‐frequency rTMS [23, 26]. Further research has revealed that combining rTMS with PE can partially mitigate the limitations associated with single‐modality exercise interventions [29], while demonstrating potential synergistic benefits on brain function in individuals with patients with SUD [30]. However, whether this combined approach can yield synergistic benefits for mood disorders in patients with MUD still lacks sufficient direct evidence. This study aims to conduct a 12‐week randomized controlled trial (8‐week intervention plus 4‐week follow‐up) to examine whether adding 10 Hz rTMS to PE yields greater benefits in improving mood disorders and reducing cravings among patients with MUD, while also uncovering the underlying neurobiological mechanisms.

## 2. Methods

### 2.1. Participants

The sample size was estimated using G ^∗^ Power software (Version 3.1.9.2), indicating that 54 participants were required to achieve a statistical power of 0.95. To account for potential participant attrition, 65 male patients with MUD were voluntarily recruited from the Xishanping Compulsory Isolation Education and Correction Center in Chongqing, China. Following the application of the inclusion and exclusion criteria, 54 individuals were selected to participate in the randomized trial.

Inclusion criteria: (1) age between 18 and 50 years; (2) currently in the rehabilitation center and having undergone withdrawal rehabilitation for more than 3 months; (3) meeting the diagnostic criteria for SUD as defined by the Diagnostic and Statistical Manual of Mental Disorders, Fifth Edition (DSM‐V). (4) voluntarily participating in this study; (5) assessed by the Physical Activity Readiness Questionnaire (PAR‐Q) [31] as eligible for moderate‐intensity aerobic exercise; (6) primary substance of use is MA. Exclusion criteria: (1) has a history of congenital (or hereditary) psychiatric illness; (2) suffering from infectious diseases such as acquired immunodeficiency syndrome (AIDS); (3) those who have received exercise or rTMS interventions within the past 3 months. This study was approved by the Southwest University Hospital Medical Ethics Committee (SHW202302231119) and obtained written informed consent from all participants in accordance with the Declaration of Helsinki.

### 2.2. Design

**Figure 1:**
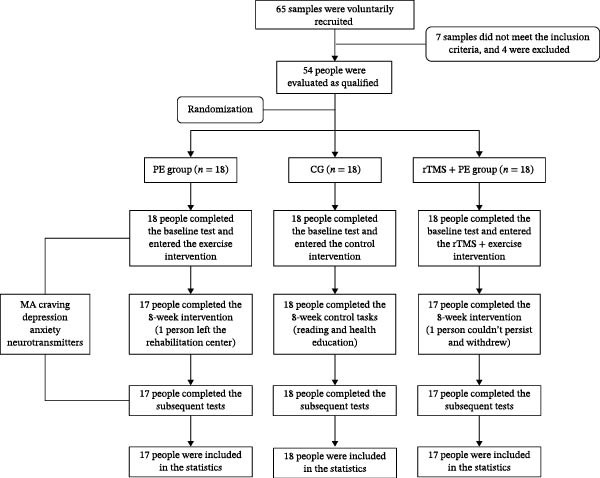
CONSORT checklist.

This study employed a randomized controlled trial design. The screened 54 participants were randomized using the random number method (numbered 1, 2, 3, 4, …), with allocation concealment implemented via the sealed envelope technique. Opaque envelopes were used to conceal the group assignment codes for each sample, with no direct display of group names on the exterior of the envelopes—only the group codes (Group A, Group B, Group C) were indicated. Among them, Group A was the PE group (“PE group”), Group B was the high‐frequency rTMS combined with PE group (“rTMS + PE group”), and Group C served as the control group (CG) (Figure 1). Both the researchers and participants were blinded to the specific group assignments. All participants were required to complete the following testing tasks and intervention/control tasks, respectively. (1) Indicator testing tasks (baseline, week 8, follow‐up period): including demographic data, MA craving, negative emotion, and blood neurotransmitter. (2) Intervention or control tasks: The PE group received an 8‐week moderate‐intensity PE intervention along with health education; the rTMS + PE group received the same exercise intervention as the PE group and high‐frequency rTMS (10 Hz), while the CG received only routine health education. It should be noted that during the experimental intervention, two participants (One participant each from the PE group and rTMS + PE group) were excluded from the final data analysis due to inability to persist with the experiment or departure from the rehabilitation center, resulting in a final sample size of 52 participants (Table 1).

**Table 1:** Demographic characteristics of participants.

Variable	PE group (*n* = 17)	rTMS + PE group (*n* = 17)	CG (*n* = 18)	*p*
Age (years)	35.94 ± 4.99	33.61 ± 6.31	35.56 ± 5.52	>0.05
Body height (m)	1.70 ± 0.05	1.72 ± 0.05	1.71 ± 0.06	>0.05
Body weight (kg)	72.83 ± 9.12	69.50 ± 9.11	71.11 ± 8.73	>0.05
Body mass index	25.64 ± 2.87	24.32 ± 2.76	24.72 ± 2.92	>0.05
Smoking history (years)	15.67 ± 7.17	14.39 ± 8.99	17.11 ± 6.46	>0.05
Drinking history (years)	9.72 ± 6.93	11.56 ± 6.89	9.83 ± 7.52	>0.05
MA usage history (years)	10.17 ± 4.96	11.06 ± 5.45	12.05 ± 4.71	>0.05
Duration of withdrawal (months)	8.61 ± 2.17	9.50 ± 2.15	9.33 ± 2.00	>0.05

#### 2.2.1. Intervention Plan

PE group: Under the guidance and supervision of professional coaches, participants are required to complete an 8‐week exercise intervention consisting of three sessions per week, each lasting 35 min. The exercise session consists of a warm‐up (3 min), treadmill running (15 min, 0% incline, at a speed of 6.5 km/h), stationary cycling (10 min), and a cool‐down (3 min), with approximately a 3‐min rest interval between the treadmill and cycling activities. The exercise prescription was developed based on previous research [32, 33], with the overall exercise intensity set at a moderate level (65%–75% of HR_max_, where HR_max_ = 206.9 − 0.67 × age) [34]. Heart rate during exercise was monitored using a heart rate monitor (Polar RCX3). Moreover, to ensure the total intervention duration per session was consistent across all groups, the exercise group received 10 min of health education prior to the workout.

rTMS + PE group: Under the operation and guidance of professionals, participants are required to undergo 10 min of rTMS (10 Hz) before completing each PE intervention (with the same content as the PE group). The rTMS protocol was implemented with reference to previous studies [23, 27] using an M‐100 Ultimate device (Yizhi, Shenzhen, China) equipped with a figure‐8 coil. The coil was positioned at a 45° angle over the left DLPFC cortex of the participant and was maintained tangentially to the scalp. The stimulation parameters were set at a frequency of 10 Hz with an intensity of 100% resting motor threshold (RMT). Each train consisted of a 5‐s stimulation delivering 50 pulses per train, followed by a 10‐s intertrain interval. The total treatment comprised 2000 pulses administered over a 10‐min duration. The left DLPFC was localized by moving the rTMS coil 6 cm anterior to the left motor area along the parasagittal line [35]. The RMT was determined by stimulating the abductor pollicis brevis representation area with the coil, and the stimulation intensity was gradually increased from 20% of the maximum output intensity until it elicited motor evoked potentials (MEPs) with amplitudes exceeding 50 μV in 5 out of 10 consecutive stimuli. This intensity value was defined as the RMT.

CG: Participants did not undergo any form of PE or rTMS intervention. They only received routine health education and correctional activities (such as reading and legal education), with each session lasting ~45 min.

#### 2.2.2. Measurement

Measurements were conducted at baseline, at the end of the 8th week, and at 1 month postintervention (follow‐up period).

1. MA craving. Self‐reported craving levels were assessed using a 10‐cm visual analog scale (VAS) ranging from “no craving at all” to “extremely intense craving.” Participants self‐rated their current craving level after viewing three randomly presented sets of images depicting paraphernalia and scenes associated with various forms of MA use [36].
2. Negative emotions. The self‐rating depression scale (SDS), revised by Liu et al. [37], was used to assess participants’ depression levels. The scale consists of 20 items rated on a 4‐point Likert scale. The total score is calculated by summing the scores of all 20 items, with scores ranging from 20 to 80 points. Higher scores indicate more severe depression levels. Additionally, the self‐rating anxiety scale (SAS), developed by Zung [38] and revised by Wang and Chi [39], was employed to assess participants’ anxiety levels. The scale consists of 20 items rated on a 4‐point Likert system. The total score is calculated by summing the scores of all 20 items and multiplying the sum by 1.25, and higher scores indicate more severe anxiety levels.
3. Neurotransmitters. Peripheral venous blood samples (5 mL) were collected from participants under fasting conditions. After collection, the blood samples were allowed to stand undisturbed for ~30 min, followed by centrifugation at 4000 rpm for 10 min. The serum was then aliquoted and stored at −80°C for subsequent analysis. The ELISA kit manufactured by Shanghai Xinyu Biotechnology Co., Ltd. (China) was employed for detection. Using a “sandwich” method, the capture antibodies were coated onto the microplate to bind target proteins in both samples and standards. Biotinylated detection antibodies then bound to the captured target proteins, followed by the binding of SABC complexes to the biotinylated detection antibodies, forming immune complexes. The optical density (OD) was measured at 450 nm using a microplate reader. The target protein concentration showed a positive correlation with the OD values, and the concentration in specimens was calculated by plotting a standard curve. This study mainly includes DA, β‐EP, and 5‐HT.

### 2.3. Statistical Analysis

Statistical analysis was performed using SPSS 23.0 software (IBM), including one‐way ANOVA and a 3 (groups) × 3 (test times) repeated measures ANOVA. Correlation analyses between variables were conducted using the R language (R Version 4.5.0). During the analysis, when the sphericity assumption was violated, degrees of freedom and *p*‐values were adjusted using the Greenhouse–Geisser correction. Post hoc comparisons were performed using the Bonferroni method, with the two‐tailed significance level set at *p* < 0.05. Data analysis was conducted from December 2024 to February 2025.

## 3. Results

### 3.1. Intervention Reduced Negative Emotions in Patients With MUD

The main effect of group on depression was not significant (*F*_2,49_ = 1.60, *p* = 0.212, *η* ^2^ = 0.06), the main effect of testing time was significant (*F*_2,98_ = 56.70, *p* < 0.001, *η* ^2^ = 0.53), and the interaction effect between testing time and group was significant (*F*_4,98_ = 13.99, *p* < 0.001, *η* ^2^ = 0.36). Simple effects analysis revealed (Figure 2a) that no significant differences were observed among the three groups at baseline (*p* > 0.05). However, both the PE group (*p* < 0.01) and rTMS + PE group (*p* < 0.01) showed significantly lower values compared to the CG at week 8. During the follow‐up period, only the rTMS + PE group remained significantly lower than the CG (*p* < 0.05). Additionally, both the PE group and rTMS + PE group showed significant reductions compared to baseline levels at week 8 (*p*  < 0.001) and during the follow‐up period (*p* < 0.01), while both groups at follow‐up showed values significantly higher than the levels at week 8 (*p* < 0.05).

Figure 2Differences in changes of depression (a) and anxiety (b) among patients at different stages. “ ^∗^” Indicates between‐group differences, with  ^∗^ representing *p* < 0.05, and  ^∗∗^ representing *p* < 0.01; “#” indicates within‐group differences, with # representing *p* < 0.05, ## representing *p* < 0.01, and ### representing *p*  < 0.001.

**Figure:**
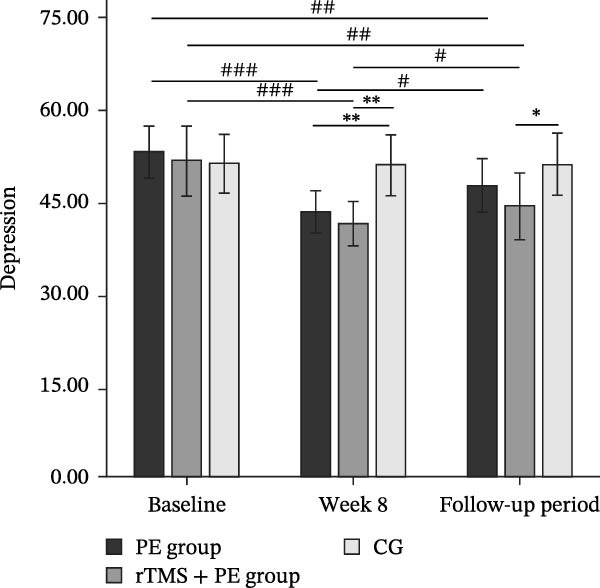
(a)

**Figure:**
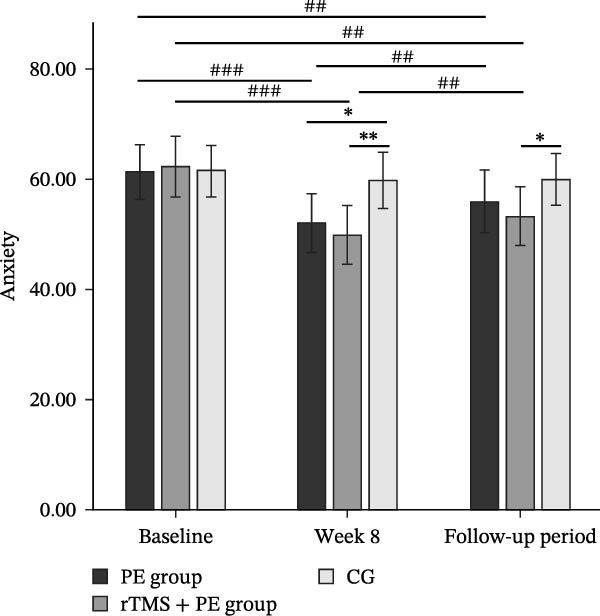
(b)

The main effect of group on anxiety was not significant (*F*_2,49_ = 1.39, *p* = 0.258, *η* ^2^ = 0.05), the main effect of testing time was significant (*F*_2,98_ = 47.85,*p* < 0.001, *η* ^2^ = 0.51), and the interaction between testing time and group was significant (*F*_4,98_ = 13.30, *p* < 0.001, *η* ^2^ = 0.35). Simple effects analysis revealed (Figure 2b) that no significant differences were observed among the three groups at baseline (*p* > 0.05). However, both the PE group (*p* < 0.05) and rTMS + PE group (*p* < 0.01) showed significantly lower anxiety levels compared to the CG at week 8. During the follow‐up period, only the rTMS + PE group remained significantly lower than the CG (*p* < 0.05). Additionally, both the PE group and rTMS + PE group showed significant reductions compared to baseline levels at week 8 (*p* < 0.001) and during the follow‐up period (*p* < 0.01), while both groups at follow‐up showed values significantly higher than the levels at week 8 (*p* < 0.05).

### 3.2. Intervention Reduces MA Craving in Patients With MUD

**Figure 3:**
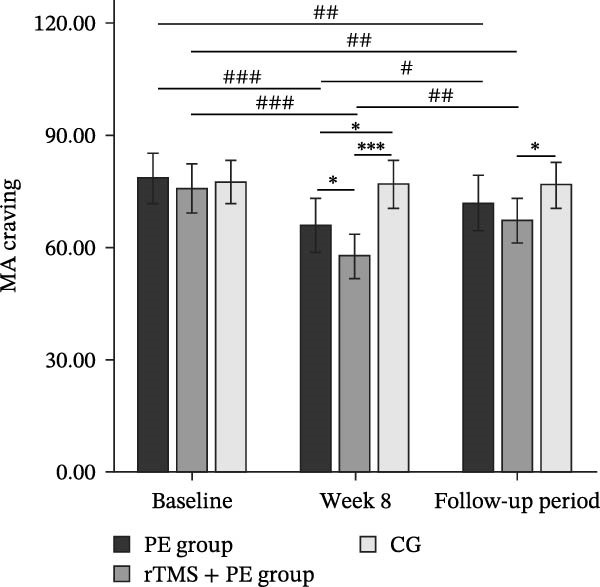
Differences in changes of MA craving among patients at different stages. “ ^∗^” Indicates between‐group differences, with  ^∗^ representing *p* < 0.05, and  ^∗∗∗^ representing *p* < 0.001; “#” indicates within‐group differences, with # representing *p* < 0.05, ## representing *p* < 0.01, and ### representing *p* < 0.001.

The main effect of group on MA craving was significant (*F*_2,49_ = 3.17, *p* = 0.046, *η* ^2^ = 0.11), the main effect of testing time was significant (*F*_2,98_ = 54.76, *p* < 0.001, *η* ^2^ = 0.51), and the interaction between testing time and group was significant (*F*_4,98_ = 19.29, *p* < 0.001, *η* ^2^ = 0.44). Simple effects analysis revealed (Figure 3) that no significant differences were observed among the three groups at baseline (*p* > 0.05). However, both the PE group (*p* < 0.05) and the rTMS + PE group (*p* < 0.001) showed significantly lower MA craving compared to the CG at week 8, with the rTMS + PE group being significantly lower than the PE group (*p* < 0.05). During the follow‐up period, only the rTMS + PE group remained significantly lower than the CG (*p* < 0.05). Additionally, both the intervention groups showed significantly lower MA craving compared to baseline levels at week 8 (*p* < 0.001) and during the follow‐up period (*p* < 0.01), while both the PE group (*p* < 0.05) and the rTMS + PE group (*p* < 0.01) were significantly higher than the levels at week 8.

### 3.3. Intervention Promotes Release of Blood Neurotransmitters in Patients With MUD

The main effect of group on DA was significant (*F*_2,49_ = 5.34, *p* = 0.008, *η* ^2^ = 0.18), the main effect of testing time was significant (*F*_2,98_ = 52.43, *p* < 0.001, *η* ^2^ = 0.52), and the interaction between testing time and group was significant (*F*_4,98_ = 15.52, *p* < 0.001, *η* ^2^ = 0.39). Simple effects analysis revealed (Figure 4a) that no significant differences were observed among the three groups at baseline (*p* > 0.05). However, both the PE group (*p* < 0.01) and the rTMS + PE group (*p* < 0.001) showed significantly higher DA levels compared to the CG at week 8, with the rTMS + PE group being significantly higher than the PE group (*p* < 0.05). During the follow‐up period, only the rTMS + PE group remained significantly higher than the CG (*p* < 0.01). Additionally, the PE was significantly higher than the baseline level at week 8 (*p* < 0.001) and during the follow‐up period (*p* < 0.01), while the rTMS + PE group was significantly higher than the baseline level at week 8 (*p* < 0.001) and during the follow‐up period (*p* < 0.001), and both groups at follow‐up showed values significantly lower than the levels at week 8 (*p* < 0.01).

Figure 4Differences in DA (a), β‐EP (b), and 5‐HT (c) levels among patients at different stages. “ ^∗^” Indicates between‐group differences, with  ^∗^ representing *p* < 0.05,  ^∗∗^ representing *p* < 0.01, and  ^∗∗∗^ representing *p* < 0.001; “#” indicates within‐group differences, with ## representing *p* < 0.01, and ### representing *p* < 0.001.

**Figure:**
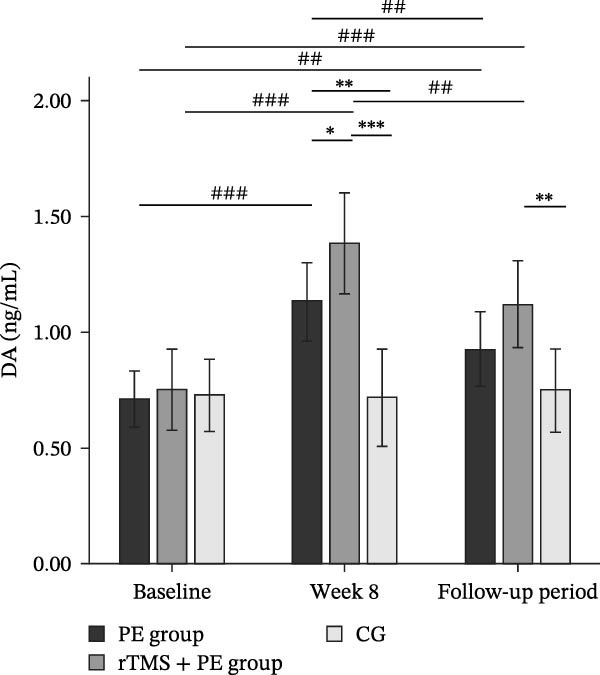
(a)

**Figure:**
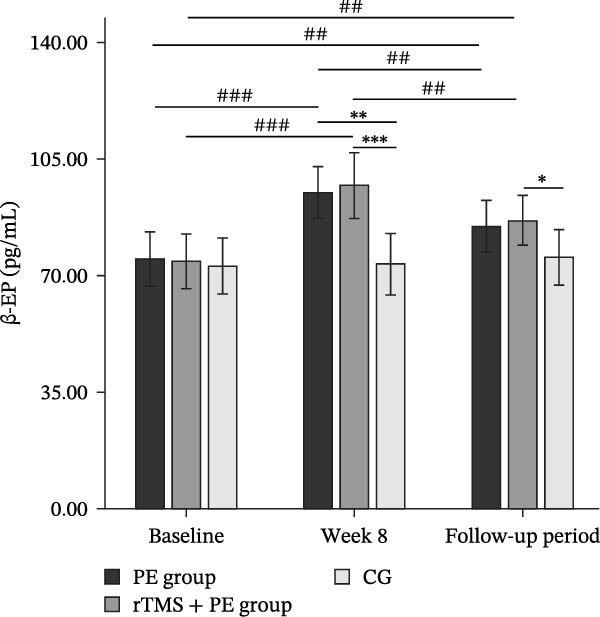
(b)

**Figure:**
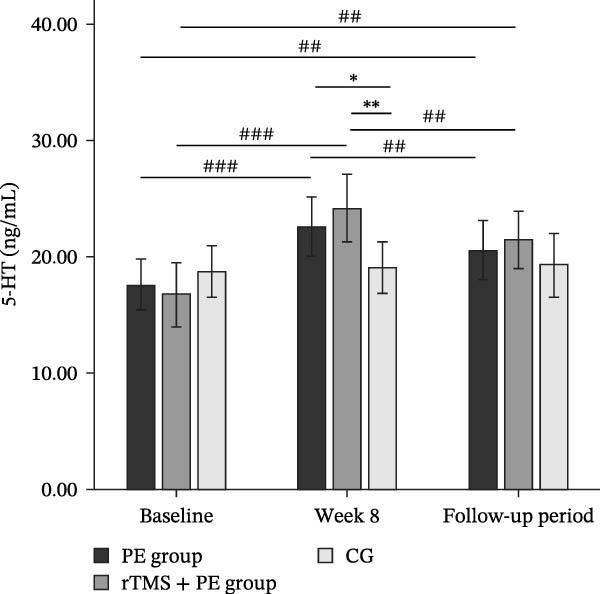
(c)

The main effect of group on β‐EP was significant (*F*_2,49_ = 3.24, *p* = 0.048, *η* ^2^ = 0.12), the main effect of testing time was significant (*F*_2,98_ = 53.03, *p* < 0.001, *η* ^2^ = 0.53), and the interaction effect between testing time and group was also significant (*F*_4,98_ = 15.08, *p* < 0.001, *η* ^2^ = 0.38). Simple effects analysis revealed (Figure 4b) that no significant differences were observed among the three groups at baseline (*p* > 0.05). However, both the PE group (*p* < 0.01) and the rTMS + PE group (*p* < 0.001) showed significantly higher values compared to the CG at week 8. During the follow‐up period, only the rTMS + PE group remained significantly higher than the CG (*p* < 0.05). Additionally, both the intervention groups showed significantly higher values at week 8 (*p* < 0.001) and during the follow‐up period (*p* < 0.01) compared to baseline levels, while both groups at follow‐up showed values significantly lower than the levels at week 8 (*p* < 0.01).

The main effect of group on 5‐HT was not significant (*F*_2,49_ = 0.68, *p* = 0.514, *η* ^2^ = 0.03), the main effect of testing time was significant (*F*_2,98_ = 45.08, *p* < 0.001, *η* ^2^ = 0.47), and the interaction effect between testing time and group was significant (*F*_4,98_ = 13.10, *p* < 0.001, *η* ^2^ = 0.35). Simple effects analysis revealed (Figure 4c) that no significant differences were observed among the three groups at baseline (*p* > 0.05), while both the PE group (*p* < 0.05) and the rTMS + PE group (*p* < 0.01) showed significantly higher values compared to the CG at week 8. Additionally, both the intervention groups showed significantly higher values at week 8 (*p* < 0.001) and during the follow‐up period (*p* < 0.01) compared to baseline levels, while both groups at follow‐up showed values significantly lower than the levels at week 8 (*p* < 0.01).

### 3.4. Correlation Analysis Among Variables

The correlation results (Figure 5) showed that after the 8‐week intervention, patients’ depression and anxiety not only exhibited significant negative correlations with DA, β‐EP, and 5‐HT, but also demonstrated a significant positive correlation with craving levels. Additionally, a significant positive correlation was observed between depression and anxiety. Specifically, the correlation coefficients ranged from −0.41 to 0.59 in the PE group (Figure 5a), from −0.44 to 0.63 in the rTMS + PE group (Figure 5b), and from −0.27 to 0.52 in the CG (Figure 5c). In comparison, the correlation coefficients in the intervention groups were greater than those in the CG.

Figure 5Correlation heatmap. (a) PE group, (b) rTMS + PE group, and (c) CG. “ ^∗^” Indicates the significance level of the correlation coefficient, with  ^∗^ representing *p* < 0.05,  ^∗∗^ representing *p* < 0.01, and  ^∗∗∗^ representing *p* < 0.001.

**Figure:**
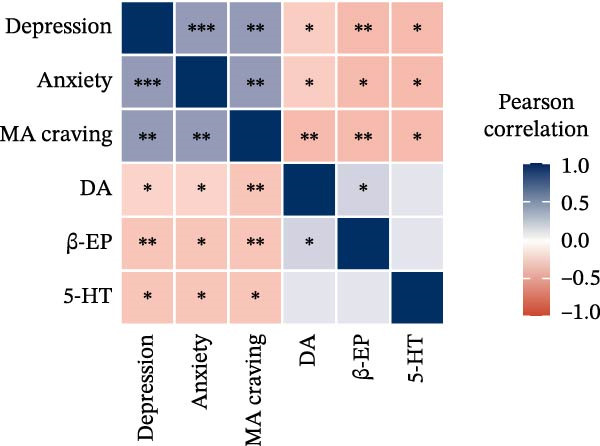
(a)

**Figure:**
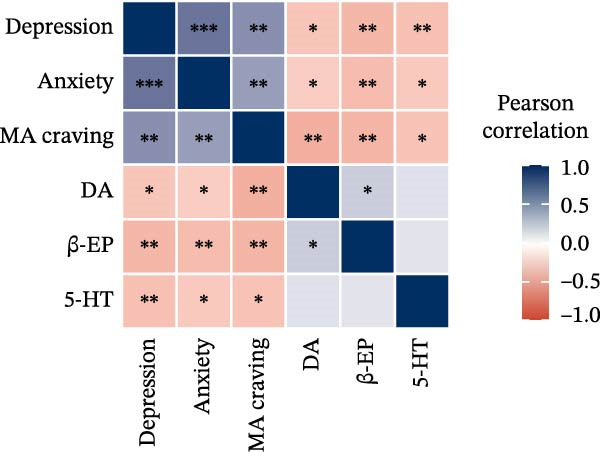
(b)

**Figure:**
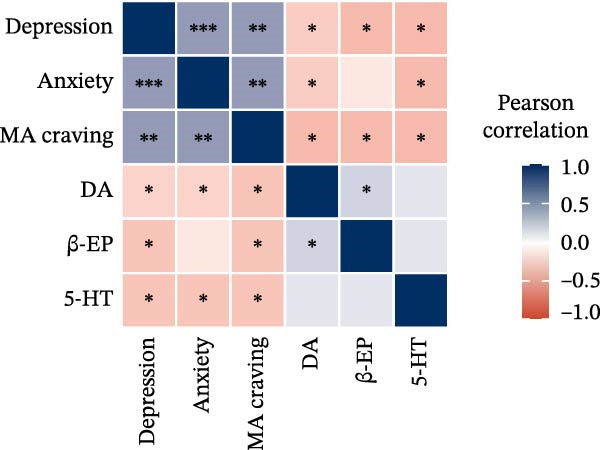
(c)

## 4. Discussions

This study mainly found that adding 10 Hz rTMS to PE was more effective in reducing negative emotions and MA craving during abstinence in patients with MUD, and the improvement in emotional regulation was associated with reduced craving and increased blood neurotransmitter levels induced by the intervention. These findings provide new evidence for addressing emotional dysregulation in patients.

In recent years, positive effects of moderate‐intensity PE [13, 14, 16] or high‐frequency rTMS [27, 40] on mood disorders in patients with MUD have been reported accordingly. A smaller number of studies have also begun to explore the synergistic benefits of combining high‐frequency rTMS with PE for withdrawal rehabilitation in patients with SUD [23, 29]. Our study revealed that both 8‐week PE and combined PE with high‐frequency rTMS interventions significantly alleviated depression and anxiety disorders in patients with MUD. However, we observed that the combined modality still showed significant differences from the CG at the 1‐month follow‐up after intervention, whereas PE alone did not demonstrate similar significant effects. This indicates that incorporating high‐frequency rTMS into PE regimens appears to better enhance or sustain the enduring intervention effects on patients’ negative emotions. However, we observed that the rTMS + PE group still showed significant differences from the CG at the 1‐month follow‐up after intervention, while there was no similar significant difference in the PE group. This indicates that incorporating high‐frequency rTMS into PE regimens appears to better enhance or sustain the enduring intervention effects on patients’ negative emotions.

The effects of interventions on mood disorders in patients with MUD may be explained by the following potential mechanisms. First, metabolic dysregulation of monoamine neurotransmitters and their receptors constitutes a significant pathogenic factor for post‐withdrawal mood disorders [10, 11], and PE can regulate neurotransmitter levels (such as DA and β‐EP) in the brain or peripheral circulation of patients [15, 41, 42], thereby simulating drug‐induced euphoria and consequently promoting the reduction of negative emotions [43]. Similarly, high‐frequency rTMS applied to the left DLPFC has also been demonstrated to effectively regulate mood disorders in patients with MUD by modulating emotional circuits (such as the prefrontal‐striatal circuit) and monoamine neurotransmitters [27, 44–46]. Interestingly, the incorporation of rTMS into PE is believed to be more conducive to alleviating withdrawal symptoms in patients with SUD [29], and this synergistic benefit may be associated with mechanisms such as dynamic regulation of neurotransmitters and their receptors, as well as enhancements in brain structure and functional connectivity [30]. Based on the findings of this study, we hypothesize that this phenomenon may be attributed to high‐frequency rTMS potentially establishing a more favorable neuroplastic environment for subsequent PE while augmenting neural excitability. Through their synergistic interaction, this combined approach appears to facilitate the enhancement and maintenance of blood neurotransmitter release levels (such as DA, β‐EP, and 5‐HT), thereby indirectly sustaining the interventional effects on negative emotions. But it should be emphasized that these viewpoints remain speculative at present, and further direct evidence is required to support them.

Second, persistent MA craving during withdrawal serves as a psychological trigger for emotional dysregulation in patients with MUD, and both PE (particularly moderate‐intensity) [14, 47, 48] and high‐frequency rTMS [24, 40] have demonstrated positive effects in reducing MA craving among this population. Our study found that not only does it support this perspective, but it also reveals that the reduction in MA craving following intervention may be associated with improvements in mood disorders and increased neurotransmitter levels. Moreover, this relationship appears to exhibit “bidirectional characteristics,” though the underlying causal mechanisms remain unclear. Research has identified that emotional state mediates the relationship between exercise intervention and MA craving [33], but there is a notable lack of similar reports concerning neurotransmitters. This study found that the incorporation of rTMS into PE demonstrates greater potential for reducing MA craving among patients, which is relatively consistent with the results of previous study [29]. It is speculated that high‐frequency rTMS may regulate neural adaptability and synaptic plasticity within the dopaminergic system [45, 49], and through synergistic coupling with PE, conjointly promotes the enhancement of synaptic efficacy in neural networks and improvement of cerebral functions [30, 50]. Therefore, we hypothesize that this benefit may more effectively activate the patient’s reward system and establish “psychological compensation,” while also facilitating the connection between central and peripheral neurotransmitters across the blood–brain barrier. This process could satisfy the pursuit of euphoria in patients with MUD, thereby further promoting the amelioration of emotional regulation circuit impairments and the reduction of craving for MA.

### 4.1. Limitations

This study has several limitations: (1) The CG only received health education, and no separate high‐frequency rTMS group or sham rTMS group was established, which may limit the ability to assess the independent efficacy of rTMS. This is because our primary aim was to investigate the additive effects of combining rTMS with PE, leading us to include only a single PE group. Future studies could incorporate separate rTMS and sham rTMS groups to better evaluate the standalone benefits of rTMS and enhance the rigor of the research design. (2) The relatively short overall intervention duration of this study (only 8 weeks) may have indirectly contributed to the lack of statistically significant intergroup differences between the rTMS + PE group and the PE group during the follow‐up period. Future studies could adopt strategies such as extending the intervention period to better highlight the sustained advantages of the combined approach. (3) Although this study identified correlations between mood disorders and blood neurotransmitter levels as well as drug craving in patients with MUD following the 8‐week intervention, the causal relationships among these variables remain unclear. Future research should employ neurobiological and cognitive neuroscience approaches to conduct more in‐depth mechanistic investigations. (4) We employed conventional high‐frequency rTMS, but a study suggests that intermittent theta‐burst stimulation (iTBS) and high‐frequency rTMS demonstrate comparable therapeutic effects on patients with MUD, with the former offering significantly reduced time requirements [40]. Consequently, whether iTBS combined with PE could yield superior therapeutic benefits for patients warrants further investigation.

## 5. Conclusion

The 8‐week moderate‐intensity PE significantly reduced depression and anxiety in patients with MUD during withdrawal, with these benefits persisting for 1 month postintervention. More importantly, we found that the model of HF rTMS combined with PE is more conducive to strengthening or maintaining the continuous impact of the intervention, which can effectively make up for the limitations of weak effect and poor sustainability of single PE. Meanwhile, the positive benefit on the emotional regulation of patients is related to the reduction of MA cravings and the increase in the release of blood neurotransmitter content after intervention. These findings suggest that in the clinical practice of treatment and rehabilitation during the withdrawal period for patients with MUD, adopting a combined intervention approach (such as 35 min of PE combined with 10 min of high‐frequency rTMS) appears to be more effective. It holds greater potential for enhancing the sustained effects of the intervention, such as in reducing negative emotions and cravings for MA.

## Author Contributions

**Kun Wang**: conceptualization, data curation, funding acquisition, resources, methodology, writing – original draft. **Yan Li**: methodology, resources, software, and writing – review and editing. **Yi Yang**: methodology, supervision, and writing – review and editing. **Tingran Zhang**: methodology, supervision, writing – review and editing. **Jiong Luo**: conceptualization, resources, supervision, funding acquisition, writing – review and editing.

## Funding

This study was supported by the National Social Science Fund of China (Grant 22BTY064) and the Chongqing Graduate Research Innovation Project of China (Grant CYB23099).

## Disclosure

All authors designed this study and contributed to, and approved, the final manuscript and its submission and publication.

## Ethics Statement

This study was approved by the Southwest University Hospital Medical Ethics Committee (SHW202302231119) following the Declaration of Helsinki, and all participants signed written informed consent.

## Conflicts of Interest

The authors declare no conflicts of interest.

## Data Availability

Data are available upon request due to privacy/ethical restrictions.
